# Genomic characterization of the NAC transcription factors, directed at understanding their functions involved in endocarp lignification of iron walnut (*Juglans sigillata* Dode)

**DOI:** 10.3389/fgene.2023.1168142

**Published:** 2023-05-09

**Authors:** Anmin Yu, Hanyu Zou, Ping Li, Xiaowei Yao, Zekun Zhou, Xu Gu, Rui Sun, Aizhong Liu

**Affiliations:** Key Laboratory for Forest Resources Conservation and Utilization in the Southwest Mountains of China, Ministry of Education, Southwest Forestry University, Kunming, China

**Keywords:** iron walnut, NAC transcription factor, lignification, endocarp (shell), gene expression

## Abstract

The NAC (NAM, ATAF1/2, and CUC2) transcription factors (TF), one of the largest plant-specific gene families, play important roles in the regulation of plant growth and development, stress response and disease resistance. In particular, several NAC TFs have been identified as master regulators of secondary cell wall (SCW) biosynthesis. Iron walnut (*Juglans sigillata* Dode), an economically important nut and oilseed tree, has been widely planted in the southwest China. The thick and high lignified shell derived endocarp tissues, however, brings troubles in processing processes of products in industry. It is indispensable to dissect the molecular mechanism of thick endocarp formation for further genetic improvement of iron walnut. In the present study, based on genome reference of iron walnut, 117 *NAC* genes, in total, were identified and characterized *in silico*, which involves only computational analysis to provide insight into gene function and regulation. We found that the amino acids encoded by these *NAC* genes varied from 103 to 1,264 in length, and conserved motif numbers ranged from 2 to 10. The *JsiNAC* genes were unevenly distributed across the genome of 16 chromosomes, and 96 of these genes were identified as segmental duplication genes. Furthermore, 117 *JsiNAC* genes were divided into 14 subfamilies (A-N) according to the phylogenetic tree based on NAC family members of *Arabidopsis thaliana* and common walnut (*Juglans regia*). Furthermore, tissue-specific expression pattern analysis demonstrated that a majority of NAC genes were constitutively expressed in five different tissues (bud, root, fruit, endocarp, and stem xylem), while a total of 19 genes were specifically expressed in endocarp, and most of them also showed high and specific expression levels in the middle and late stages during iron walnut endocarp development. Our result provided a new insight into the gene structure and function of *JsiNAC*s in iron walnut, and identified key candidate *JsiNAC* genes involved in endocarp development, probably providing mechanistic insight into shell thickness formation across nut species.

## Introduction

Transcription factors (TFs) are critical regulators by activating or repressing the expression of targeted genes and widely participated in regulation of plant growth and development, stress response and metabolism processes (Riechmann et al., 2000; Wray et al., 2003; Puranik et al., 2012). The NAC genes, one of the largest plant specific TF families, were named according to the initial discoveries of genes NAM (no apical meristem) in Petunia, *ATAF1/2* (transcription activation factors) and *CUC2* (cup-shaped cotyledon) in *A. thaliana* (Arabidopsis) (Olsen et al., 2005). Typically, the N-terminal DNA binding domains of NAC TFs are conserved and can be divided into five functionally distinct sub-domains (designated as A-E), while the C-terminal regions are highly divergent. The divergence of domains in the C-terminal regions among NAC members often resulted in their diverse regulatory activities by binding different cis-elements (Ooka et al., 2003).

Previous studies have found that NAC TFs were functionally involved in diverse biological processes in plant growth and development, such as hormone signal transduction, leaf senescence and fruit development, lateral root development, and abiotic and biotic stress response (Weir et al., 2004; Kim et al., 2012; Yang et al., 2014; Ma et al., 2018; Diao et al., 2020). In particular, many studies have revealed that NAC members play crucial roles in regulating the processes of xylogenesis in different plants, such as wood formation, fiber development, and seed coat lignification (Ohtani et al., 2011; Zhao et al., 2014; Xia et al., 2019). Several NAC TFs have been described as the first-layer master regulators that control the biosynthesis of secondary cell wall (SCW) in both herbaceous and woody plants (Nakano et al., 2015). It is well known that the primary components of SCW are lignin, cellulose and hemicellulose, which provide remarkable rigidity and strength to support the space extension for cell and plant body growth (Zhong et al., 2019). The NAC-mediated transcriptional network of SCW biosynthesis is cell type specific with three mainly distinct sister groups: Vascular Related NAC-Domain (VND), Secondary Wall-Associated NAC Domain Protein (SND), and NAC Secondary Wall Thickening Promoting Factor (NST) (Mitsuda et al., 2005; Zhong et al., 2007; Ohashi-Ito et al., 2010; Zhou et al., 2014; Tan et al., 2018). Specifically, the VND group members (such as VND6 and VND7) have been demonstrated to be key regulators for vascular vessel formation in Arabidopsis (Kubo et al., 2005); the SND group members (such as SND1-5) were thought to be critical transcriptional activators of SCW biosynthesis in the fibers of Arabidopsis (Zhong et al., 2006). In the loss-of-function mutant of *AtVND3*, SCW thickening was largely attenuated, leading to a collapse of xylem biosynthesis (Zhou et al., 2014). Since SCW thickening occurs widely in diverse tissues such as stem, seedpod and anther endothecium, NST group members often invovled in the morphogenesis of many tissues via influencing SCW formation and thickening. For example, *AtNST1* and *AtNST2* are highly expressed in the anther tissue, regulating SCW thickening of the anther endothecium in Arabidopsis (Mitsuda et al., 2005). *CpNST1* gene was specifically expressed in seed coat tissue of pumpkin, regulating SCW biosynthesis and thickening of pumpkin seed coat (Lyu et al., 2022). Several studies have found that the homologs of *NST1* were specifically expressed in endocarp tissues, which was critical in promoting the lignification process of endocarp in peach, apricot, and Arabidopsis (Dardick et al., 2010; Zhang et al., 2017). In addition, a recent study found that *AtXND1* (*Xylem NAC Domain1*), was functionally involved in switching the differentiation of xylem vessel cells by regulating terminal SCW biosynthesis and programmed cell death in Arabidopsis (Zhong et al., 2021). Taken together, studies have revealed that the functions of NAC members often are involved in regulating SCW biosynthesis and lignification processes in diverse tissues though they are probably variable in different species or tissues. Thus, identification and characterization of potential NAC members involved in SCW biosynthesis and lignification processes are necessary to understand the development and differentiation processes for a given tissue.

Walnut is an important nut and woody oil tree, widely planted all over the world because of its rich nutritional compounds in seeds and the valuable wood of its stems (Martínez et al., 2010). Usually, the thickness or thinness of walnut endocarp is often of a critical economic trait, for it is tightly associated with the breakdown and discardness of the shell prior to the processing process of products. In particular, the iron walnut (*J. sigillata* Dode) with thick endocarp has been widely planted in southwestern mountain areas of China because of its strong adaptability to diverse soil and climate conditions (Ning et al., 2020). Dissection of the potential molecular mechanism underlying the formation of thick endocarp is important for genetic improvement to create new iron walnut varieties with thin shell. Here, we identified and characterized genome-wide NAC members based on the genome data of iron walnut. We screened and sorted out endocarp-specifically expressed NAC genes and candidates involved in regulating the formation of thick endocarp by comparative transcriptomes analyses among diverse tissues and different stages of endocarp development. This study provides basic data and useful clues to understand the molecular mechanism of thick endocarp formation in iron walnut.

## Material and methods

### Plant materials

The ca. 100 years old iron walnut individuals of varieties “Dapao” and “Tie” planted in a plantation of Guangming town (25°40′N, 100°1′E), Dali Autonomous Prefecture, Yunnan Province, China, were selected as study materials in this study. Fresh fruits were collected every 30 days after pollination (DAP) from 1st April to 16th October, in 2020. Thirty samples from iron walnut fruits at each stage were collected from three biological replicates (three independent trees per replicate). The fruits were dissected immediately after harvest, and endocarp tissues were flash-frozen in liquid nitrogen, and then stored at −80°C for RNA extraction. According to the progression of iron walnut fruit development recently published (Yu et al., 2023), the fruits at 60, 90, and 120 DAP were used to represent the initiation stage of endocarp lignification, the endocarp hardening stage, and the maturation stage for transcriptome sequencing (using “Dapao,” a cultivated iron walnut variety with thin shell) and qRT-PCR analysis of gene expression (using “Tie,” a wild iron walnut variety with thick shell).

### Genome-wide identification and characterization of the *JsiNAC* genes

Protein sequences and genome annotation file of *J. sigillata* were downloaded from the GigaScience Database (http://gigadb.org/dataset/100693) (Ning et al., 2020). Then, the Hidden Markov Model (HMM) of the NAM domain (PF02365) was obtained from the PFAM database (pfam.xfam.org), and a hmm search was performed using the HMMER 3.2.1 program with an E-value value of 1e^−10^ against all amino acid sequences in the genome of *J. sigillata* (Finn et al., 2011). Subsequently, the conserved domains of all hits were further individually analyzed with the InterProScan (www.ebi.ac.uk/interpro/search/sequence/) and SMART (https://smart.embl.de) databases (Letunic et al., 2012; Jones et al., 2014). Finally, the tools on the ExPASy website (http://www.expasy.org/tools/) were used to investigate the number of amino acids, molecular weight (MW), theoretical isoelectric point (pI), aliphatic amino acid index and protein hydrophobicity values (Duvaud et al., 2021).

### Phylogenetic analysis and classification of the NAC gene family in iron walnut

To infer the evolutionary conservation of the NAC gene family between *J. sigillata* and the most popular species in the genus *Juglans*, common walnut *J. regia*, the amino acid sequences of *J. regia* NAC family genes were retrieved from NCBI according to the gene ID listed in a previous study (Kang et al., 2021). To comprehensively compare the differences of NAC family genes between *J. sigillata* and *J. regia*, the multiple sequence alignment and phylogenetic analysis were performed by ClustalW and MEGA 11, respectively (Thompson et al., 1994; Hall, 2013). Furthermore, to investigate the origin and classification of *J. sigillata*, the amino acid sequences of *NAC* genes of Arabidopsis were also downloaded from the TAIR database (http://www.arabidopsis.org). All genes from the *J. sigillata, J. regia*, and Arabidopsis were merged together and constructed a phylogenetic tree was constructed using the neighbor-joining (NJ) method in MEGA 11 (Hall, 2013). Then, interactive Tree of Life (iTOL) (https://itol.embl.de/tree) was used to visualize and optimize the phylogenetic tree (Letunic and Bork, 2021).

### Conserved domain, gene structure and motifs of *NAC* genes in iron walnut

The gene structure characteristics of the *NAC* genes in iron walnut were identified by the Gene Structure Display Server 2.0 (GSDS, http://gsds.cbi.pku.edu.cn/) (Hu et al., 2015). The conserved motifs of these NAC protein sequences were elucidated using the online MEME (Multiple EM for Motif Elicitation) tool (version 5.1.1, https://meme-suite.org/) on the basis of the following parameters: the maximum number of motifs was 15, with a minimum width of 50 and a maximum of 100 and other parameters were set to default (Bailey et al., 2015). Finally, TBtools software was used to draw a picture through merging the evolution tree of *NAC* genes in iron walnut with gene structure and motif composition (Chen et al., 2020).

### Chromosomal distribution and collinearity analysis

The relative locations of all *NAC* genes on different chromosomes of *J. sigillata* were obtained from the annotation information (GFF3) of the reference genome, and visualized using TBtools software. The duplication pattern of all *NAC* genes was analyzed using the BLASTP program and Multiple Collinearity Scan toolkit (MCScanX), and then visualized using TBtools software (Wang et al., 2012; Chen et al., 2020).

### Analysis of the expression profiles of *NAC* genes in iron walnut based on RNA-seq

To investigate the expression pattern of *NAC* genes in distinct tissue types (bud, root, fruit, endocarp, stem xylem region) as described by Martinez-Garcia et al (2016), the RNA-seq clean data were downloaded from NCBI (BioProject: PRJNA291087), then mapped to the *J. sigillata* reference genome (Ning et al., 2020). The process of RNA-seq analysis was implemented in our in-house pipeline (Yu et al., 2023). The expression levels of *JsiNAC* genes were extracted and filtered (the sum FPKM values were greater than 1), then visualized using a heatmap via the R program of pheatmap (Hu, 2021). The expression patterns of *NAC* genes during iron walnut endocarp development were acquired from our recently published transcriptome data of “Dapao” (BioProject: PRJNA928586). To further identify genes encoding TFs in iron walnut, all genes were queried against the Plant Transcription Factor Database (PlantTFDB) with the E-values threshold of 1E-05 (Jin et al., 2017). The FPKM values of all *NAC* genes were normalized by Z-score and displayed in the heatmaps as described previously (Hu, 2021). Gene expression patterns of common differentially expressed genes in the endocarp transcriptomes of “Dapao” at P1 (60 DAP), P2 (90 DAP), P3 (120 DAP) stage were used to predict targets of iron walnut NAC TFs by the R package GENIE3 (Huynh-Thu et al., 2010; Love et al., 2014). The co-expression network was displayed using Cytoscape v3.9.1 (Shannon et al., 2003; Huerta-Cepas et al., 2019).

### RNA isolation and quantitative real-time PCR (qRT-PCR)

Total RNA of iron walnut “Tie” endocarp at T1, T2, T3 three different developmental stages (60, 90, and 120 DAP) was extracted, respectively, and the cDNA was synthesized with 1 μg of total RNA using the TransScript All-in-One First-Strand cDNA Synthesis SuperMix for qRT-PCR kit (TransGen Biotech, Beijing, China). The primers were designed using the NCBI Primer-BLAST tool (https://www.ncbi.nlm.nih.gov/tools/primer-blast/) and were shown in Supplementary Table S1. qRT-PCR amplifications were carried out with the PerfectStart Green qPCR SuperMix kit (TransGen Biotech, Beijing, China). The reactions were performed as follows: 95°C for 3 min, followed by 40 cycles of 95°C for 5 s, 60°C for 20 s, and 72°C for 15 s. Each gene and each experiment was tested by three biological replicates with three technical replicates. *Jsi*ACTIN was used as the reference gene. Relative expression levels were determined by the 2^−ΔΔCt^ method (Livak and Schmittgen, 2001).

## Results

### Genome wide identification of the NAC genes in iron walnut

Based on the reference genome data of iron walnut (*J. sigillata*, NCBI: txid224355) we used HMMER as the search engine and obtained 118 NAC candidates. After all candidate sequences were filtered using InterPro and SMART to confirm that they contained more than three complete structural domains, 117 were considered as *JsiNAC* genes and designated as *JsiNAC*1 to *JsiNAC*117 (Supplementary Table S2) The JsiNAC members varied markedly in terms of protein sequence length, from 103 (*JsiNAC50*) to 1,264 (*JsiNAC80*) amino acids (aa), and the molecular weights of the proteins ranged from 12.21 (*JsiNAC50*) to 139.88 kDa (*JsiNAC80*). The theoretical pI values ranged from 4.55 (*JsiNAC91*) to 9.50 (*JsiNAC85*), 82 *JsiNAC* members with pI values less than 7 were classified as acidic proteins, and 35 *JsiNAC* genes with pI values greater than 7 were classified as basic proteins. The protein hydrophobicity values were negative for all *JsiNAC* genes except for *JsiNAC15* (0.175), which was positive, indicating that most JsiNAC are hydrophilic proteins (Supplementary Table S2). According to the above analysis, these *JsiNAC* genes were highly diverse in both sequence length and physicochemical properties. Furthermore, we constructed a phylogenetic tree of 121 *JrNAC* genes from common walnut (*J. regia*) and the 117 *JsiNAC* genes identified in this study, as shown in Supplementary Figure S1 (Kang et al., 2021). The results revealed that a total of 105 *NAC* genes were highly conserved homologous between iron walnut and common walnut, and only 12 *JsiNAC* genes and 16 *JrNAC* genes were uniquely presented in iron walnut and common walnut, respectively (Supplementary Figure S2).

### Gene structure and conserved motif analysis of the *JsiNAC* gene family and analysis of the conserved structural domain

**FIGURE 1:**
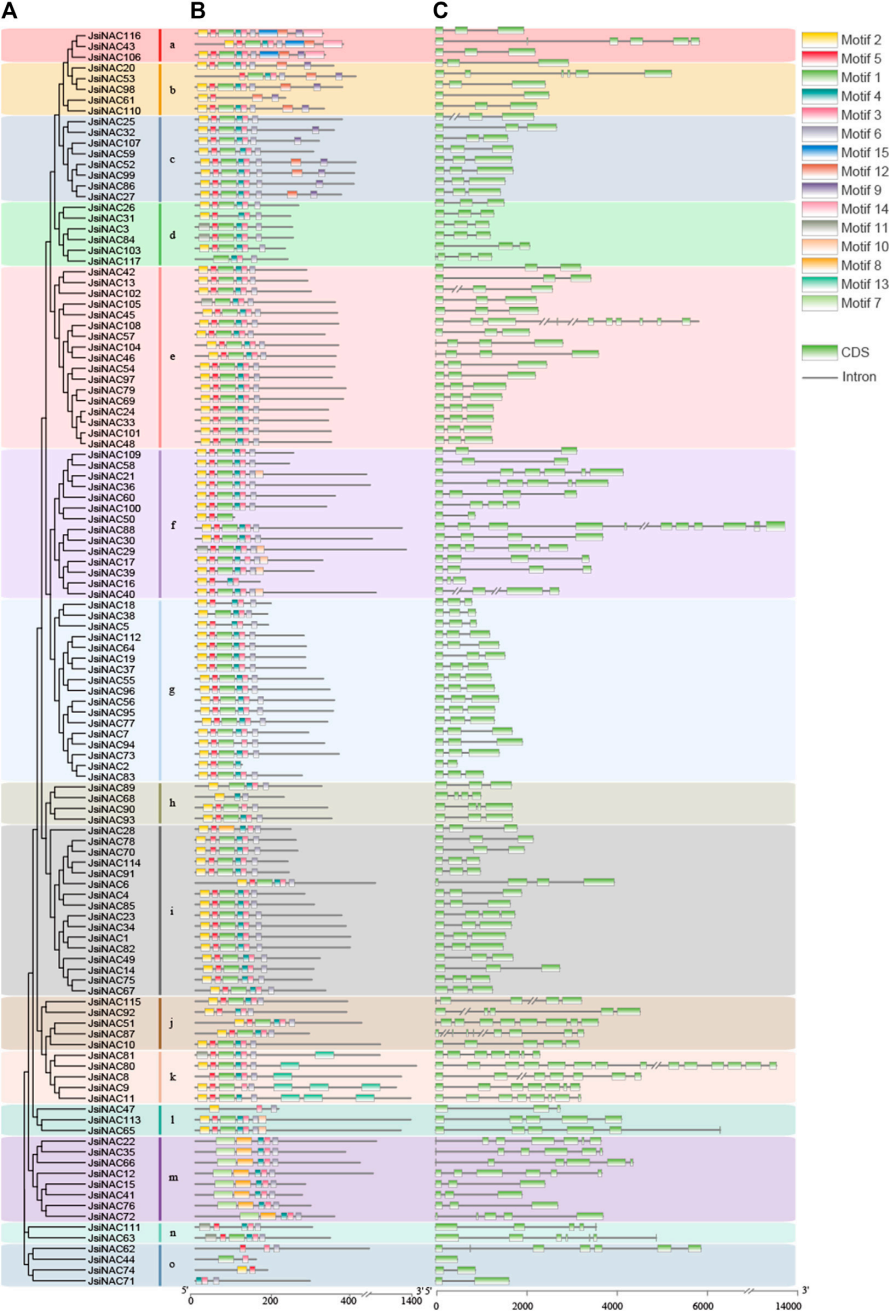
Gene structure and conserved protein motif structure of *JsiNAC* genes. **(A)** A rootless phylogenetic tree was constructed based on the full-field sequence of JsiNAC proteins using the NJ method with 1,000 bootstrap replicates. These subgroups of NAC proteins are represented by different colored blocks. **(B)** Prediction of conserved JsiNAC protein motifs by the MEME program, with different colored boxes representing different patterns and black lines representing non-conserved sequences. **(C)** Intron/exon structure of *JsiNAC* genes is shown using TBtools software, black lines represent introns and green boxes represent exons. The length of exons and introns is shown proportionally, and the amino acid sequence of the C-terminus of the NAC protein is not fully displayed.

To further understand the gene structural diversity and similarity of *JsiNAC* genes, we investigated their intron/exon organization and conserved motifs based on multiple protein sequence alignment. All *JsiNAC* genes were divided into 15 subgroups (subgroups a-o) according to their protein sequence, gene structure and motif composition (Figure 1A). We found that the number of exons in all *JsiNAC* genes ranged from 1 to 14, and the number of introns ranged from 1 to 13, exhibiting significant differences in intron/exon numbers among different genes. *JsiNAC80* had the largest number of introns (13) and exons (14), followed by *JsiNAC88*, which consisted of 11 exons and 10 introns. The exon numbers of *NAC* members in the k subgroup, in which *JsiNAC80* was located was more than 7 (Figure 1C). We found that *JsiNAC* members in different subgroups also had similar gene structures; for example, *JsiNAC3/42/55/77/83/99* were in different subgroups (d, e, g and c), but their gene structures were similar, all containing three exons and two introns (Figure 1C). Majority of *JsiNAC* members (71/117) contained three exons, which may be related to a relatively conserved gene structure in the NAC family.

To further analyze the functional regions of JsiNAC proteins*,* conserved motifs of all *JsiNAC* members were identified by MEME software. In total, 15 different motifs were identified in all *JsiNAC* genes (Figure 1B), and the sequence details of each motif were shown in Supplementary Table S3. As expected, most of the closely related genes had similar motif types and arrangements in each subgroup of Figure 1A. Motifs 1-6 were present in nearly all JsiNAC proteins. However, some motifs were only present in few subgroups. For example, motif 7 and 8 were especially distributed in subgroup m, motif 9 and 12 were shared by the *NAC* members of subgroups a, b and c, motif 10 existed in some of the members in subgroups, f and l, and motif 14 and 15 only existed on *JsiNAC43/106/116* in subgroup a. In contrast, the *NAC* members in subgroup o all contained two or three conserved motifs, such as *JsiNAC62* only containing motif 2, 3, and 6. Similar to the shared gene structure of *NA*C genes in different subgroups, the motif compositions of *NAC* genes were also similar among different subgroups, such as *JsiNAC42/33/56/95/96*, which were in subgroups e and g. The results were generally consistent with those mentioned in *J. regia* (Kang et al., 2021). For instance, the *JsiNAC* genes in the c and m subgroups were corresponded to the members of IV and XVI subgroups in the previous study of *J. regia*, which showed a high degree of consistency in gene structure and conserved motif composition (Kang et al., 2021) (Supplementary Figure S1).

Subsequently, the full-length amino acid sequences of 117 *JsiNAC* genes were used for structural domain identification. As shown in the Supplementary Figure S3, the iron walnut NAC TFs contained five NAC domains (domain A-E), and they were displayed according to the order in Figure 1. However, some *JsiNAC* genes including *JsiNAC28*, *50* and *74*, which belongs to subgroups i, f, and o, respectively, lacked structural domains D and E. Additionally, *JsiNAC* members of *JsiNAC68* in subgroup h lacked the C and E domains, *JsiNAC117* in subgroup d and *JsiNAC105* in subgroup e lacked the B domain, *JsiNAC2* in the g subgroup lacked the E domain, and *JsiNAC61* in subgroup b lacked the C and D domains. The domains of *NAC* members within the same subgroup were relatively conserved. However, the domain sequences were variable among different subgroups. For example, the amino acid sequences of B domains in subgroup m were differed considerably from other subgroups, which was presumably related to the different functions of *JsiNAC* genes in subgroup m.

### Classification of *JsiNAC* genes through phylogenetic analyses using *A. thaliana NAC* genes

**FIGURE 2:**
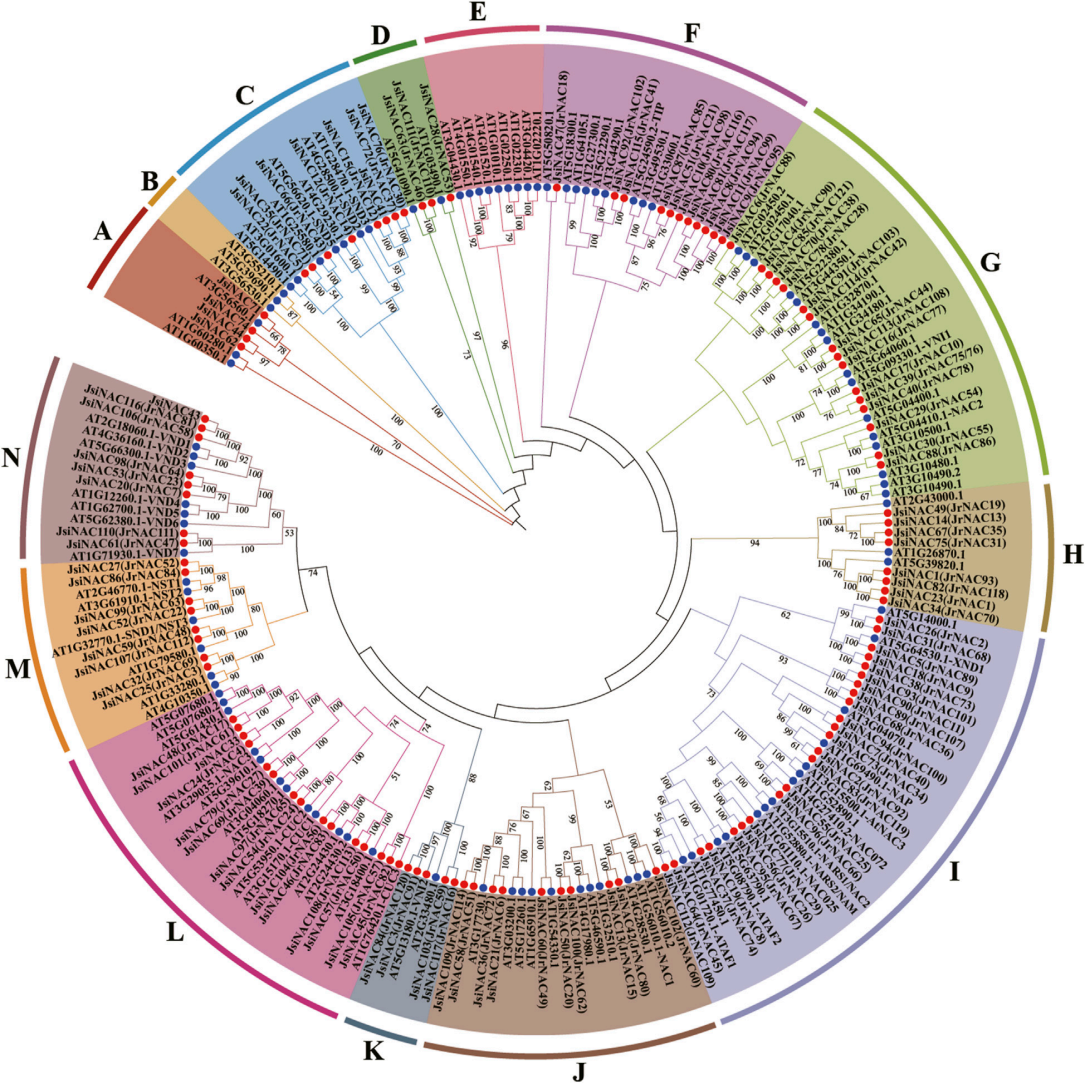
Phylogenetic tree of *NAC* genes in *A. thaliana* and *J. sigillata*. The *JsiNAC* and *AtNAC* genes are followed by red and blue circles, respectively. They are divided into 14 subfamilies according to the subgroups of *A. thaliana*. Phylogenetic trees were constructed using the neighbor-joining (NJ) method with 1,000 bootstrap replicates. These 14 protein subfamilies were represented by different colors, and their names were marked with upper case letters.

To explore the evolutionary relationships of the *JsiNAC* gene family, an unrooted phylogenetic tree was constructed using the amino acid sequences of *JsiNACs* (117 genes) and *AtNACs* (105 genes) members, and these *NAC* genes were divided into 14 subfamilies (A-N) (Figure 2). Among them, subfamily I had the most *JsiNAC* genes with 23 members, followed by subfamily L with 14 genes. The subfamily D with the fewest gene numbers (only 3 genes), and subfamilies B and E possessed only *AtNAC* genes. Notably, homologous genes with the same function had a strong tendency to be clustered into the same subfamily. The *AtSND* genes related to xylem vessel development were clustered in subfamily C with 7 *JsiNACs* genes (Zhong et al., 2006), such as *JsiNAC15*, *JsiNAC66*, and *JsiNAC72*. In subfamily I, the *JsiNAC56* and *JsiNAC95* were clustered closely with ovule integument development related genes *NARS1/2* (*AT3G15510.1/AT1G52880.1*), while *JsiNAC55* and *JsiNAC96* were clustered closely with the leaf senescence related genes *AtNAC072* (*AT4G27410.2*) (Kunieda et al., 2008; Ma et al., 2018). We noted that *JsiNAC5, JsiNAC18* and *JsiNAC38* were closely related to the negative regulatory of xylem vessel genes *AtXND1*(*AT5G64530.1*) (Zhong et al., 2021). *AtNST* genes and 8 *JsiNAC* genes were commonly existed in subfamily M, while *AtVND* genes and other eight *JsiNAC* genes were clustered into subfamily N (Figure 2). In addition, *NAC* genes from *J. regia* were also involved in this phylogenetic tree, and the subfamily clustering of the two walnut species were highly consistent. For example, the *JsiNAC* and *AtNAC* members in subfamily C in this phylogenetic tree were consistent with the subfamily *ONAC003* in the previous study of common walnut (Kang et al., 2021). These similarities between different studies suggested that this NAC phylogenetic analysis is accurate and reliable.

### Chromosomal localization and collinearity analysis of the *JsiNAC* gene family

Chromosome distribution analysis of the walnut genome revealed that 115 of the 117 *JsiNAC* genes were unequally distributed among the 16 chromosomes, while *JsiNAC116* and *JsiNAC117* were found on Scaffold268 and Scaffold538, respectively. Chromosome (Chr) 2 contained the most *JsiNAC* genes (15, 12.8%), followed by Chr1 (11, 9.4%), and Chr16 contained the fewest *JsiNAC* genes (2, 1.7%). In addition, a total of 4 (3.4%), 12 (10.2%), 4 (3.4%), 10 (8.5%), 10 (8.5%), 6 (5.1%), 7 (5.9%), 6 (5.1%), 3 (2.6%), 6 (5.1%), 9 (7.7%), 3 (2.6%), 7 (5.9%) genes were located on Chr3, 4, 5, 6, 7, 8, 9, 10, 11, 12, 13, 14, 15, respectively (Table 1; Supplementary Figure S4). In addition, genes on the same chromosome mostly were belong to different subfamilies of the phylogenetic tree. For example, all the seven *JsiNAC* genes in the N subfamily, clustered with *VNDs* on the phylogenetic tree, were distributed across six different chromosomes (Chr2, 5, 6, 7, 13, 15).

**TABLE 1:** Distribution and ratio of *JsiNAC* gene on 16 chromosomes.

Chromosome name	Number of *JsiNAC* genes	Percentage value (%)
Chr 01	11	9.4
Chr 02	15	12.8
Chr 03	4	3.4
Chr 04	12	10.2
Chr 05	4	3.4
Chr 06	10	8.5
Chr 07	10	8.5
Chr 08	6	5.1
Chr 09	7	5.9
Chr 10	6	5.1
Chr 11	3	2.6
Chr 12	6	5.1
Chr 13	9	7.7
Chr 14	3	2.6
Chr 15	7	5.9
Chr 16	2	1.7

**FIGURE 3:**
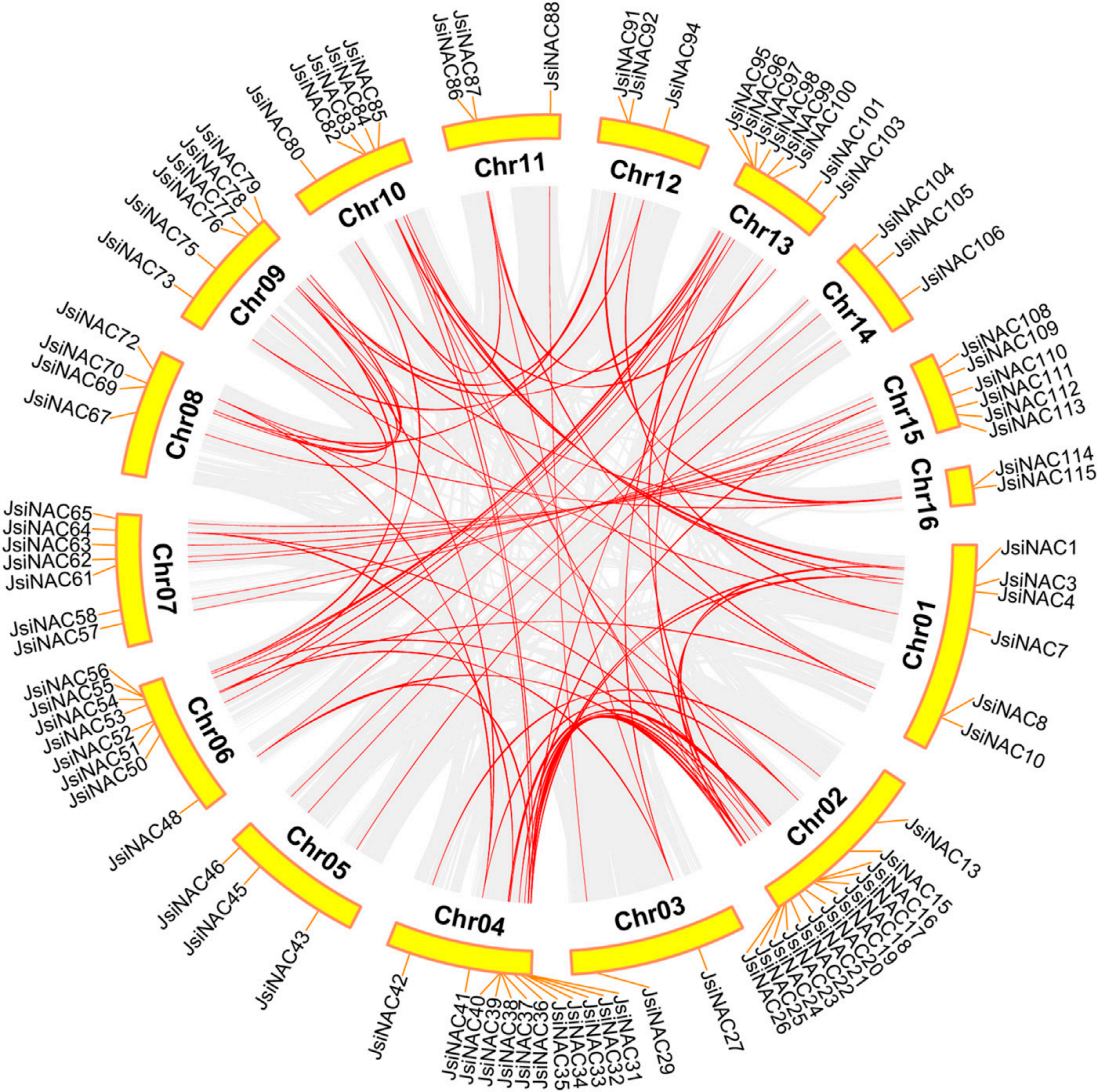
Collinearity analysis of *JsiNAC* genes. The gray line indicates all synchronized blocks in the *J. sigillata* genome and the red line indicates duplicated NAC gene pairs.

Gene duplication events were associated with plant evolution, and tandem and segmental duplications were sources of gene family expansion and genomic complexity (Cannon et al., 2004). Synteny analysis revealed that 109 out of 117 *JsiNAC* were syntenic to genes in the iron walnut genome. There were 6 groups of tandemly duplicated *JsiNAC* genes (*JsiNAC8/9/10/11*, *JsiNAC29/30*, *JsiNAC55/56*, *JsiNAC80/81*, *JsiNAC89/90*, *JsiNAC95/96*), and these genes were distributed on 6 different chromosomes, Chr1, 3, 6, 10, 12 and 13, respectively. A total of 80 *JsiNAC* orthologous gene pairs (There are 96 *JsiNAC* members in the collinearity region, and there are repeated collinearity among them) arose from genome duplication events, such as *JsiNAC56*-*JsiNAC95* and *JsiNAC55-JsiNAC96* (Figure 3). These results suggested that some *JsiNAC* genes may have arisen through gene duplication events, and segmental duplication events played a major role in the expansion of the iron walnut *NAC* gene family.

### Analysis of the tissue specific and endocarp development specific expression pattern of *JsiNAC* genes

**FIGURE 4:**
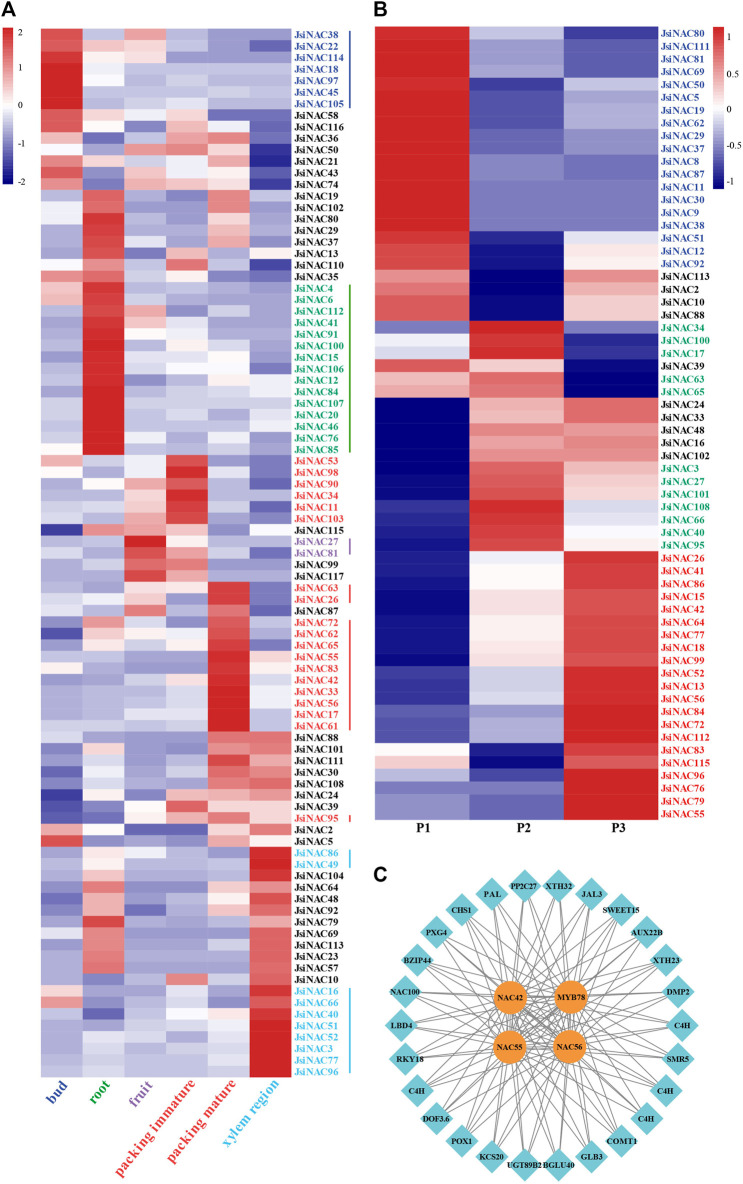
Identification of *JsiNAC* related to endocarp development. **(A)** Expression patterns of 91 *JsiNAC* genes with the sum of FPKM values greater than 1 in five different tissues of walnut. The five tissues include bud, root, fruit, endocarp, and stem xylem. **(B)** Expression levels of 62 *JsiNAC* genes with FPKM values greater than 1 in the endocarp of iron walnut at three different developmental periods, P1, P2, and P3 represented 60, 90, and 120 DAP of iron walnut fruits, respectively. The blocks with *red* or *blue* color represent the up or downregulated genes. **(C)** The co-expression network based on JsiNAC TFs and their target genes.

To better understand the gene expression pattern of the 117 *JsiNAC* genes in the growth of walnut, we analyzed RNA-seq data from five different tissues, bud, root, xylem region in stem, fruit, immature and mature endocarp (Martinez-Garcia et al., 2016). A total of 91 *JsiNAC* genes with the sum FPKM values greater than 1 were selected for heat map analysis (Figure 4A; Supplementary Table S4). As shown in the heat map, seven *JsiNAC* genes were specifically expressed in the bud, fifteen *JsiNAC* genes were specifically expressed in the root, ten *JsiNAC* genes were highly expressed in the xylem region, and only two *JsiNAC* genes were specifically expressed in the fruit. The results revealed that a total of 19 *JsiNAC* genes showed specific high expression levels in the immature and/or mature endocarp of walnut, and the FPKM values of *JsiNAC55* and *JsiNAC56* were high in the mature endocarp tissue (more than 350 FPKM).

To further explore the molecular mechanisms and regulatory processes of *JsiNAC* genes in walnut endocarp, we applied RNA-seq analysis of iron walnut endocarp at 60, 90, and 120 DAP with three biological replicates in our previous study, which represented the endocarp rapid growth (P1), hardening (P2), and mature (P3) stage, respectively (Yu et al., 2023). Furthermore, a total of 2023 TFs from 57 gene families were found in the iron walnut transcriptomes, and the largest number of TFs was the bHLH family with 194 genes, followed by the MYB and NAC families containing 192 and 117 TFs, respectively (Table 2). For these 117 NAC TFs, 42 were differentially expressed genes (DEGs) during endocarp development. To comprehensively investigate the expression pattern of *JsiNAC* genes from endocarp rapid growth stage to mature stage, 62 *JsiNAC* genes (including above mentioned 42 DEGs) with total FPKM values >1 in all three samples were selected for gene expression pattern analysis (Figure 4B; Supplementary Table S5). The results showed that 19 *JsiNAC* members were specifically expressed in the early development stages of iron walnut endocarp (Figure 4B in *blue*). A total of 12 *JsiNAC* genes showed higher levels of expression only in the middle stage of endocarp development (Figure 4B in dark *green*), and 21 *JsiNAC* genes were specifically highly expressed in the late stage (Figure 4B in *red*). It was notable that most of the endocarp-specific expression genes identified in Figure 4A were also highly expressed in the middle and/or late stage of iron walnut endocarp. To identify genes significantly related to lignified shell formation and the target genes of essential NAC TFs, a co-expression network was constructed based on the DEGs in the transcriptomes of P1, P2, and P3. As shown in Figure 4C, *JsiNAC42*, *JsiNAC55*, *JsiNAC56*, and *JsiMYB78* shared the maximum number of nodes with genes involved in SCW biosyntheses, such as *JsiPAL (phenylalanine ammonia-lyase, OF26774)*, *JsiCOMT1 (caffeic acid 3-O-methyltransferase, OF12093),* and *JsiXTH23 (xyloglucan endotransglucosylase/hydrolase protein 23, OF15252).* Hence, it appears likely that *JsiNAC* genes were important for endocarp formation and lignification via regulating essential genes involved in phenylpropanoid biosynthesis pathway.

**TABLE 2:** Top 10 transcription factors families in iron walnut transcripts.

Transcription factor family name	Number of genes
bHLH	194
MYB	192
ERF	161
C2H2	129
NAC	117
WRKY	88
bZIP	84
GRAS	73
MYB_related	73
HD-ZIP	56

### Detection of *JsiNAC* gene expression by quantitative real-time PCR

**FIGURE 5:**
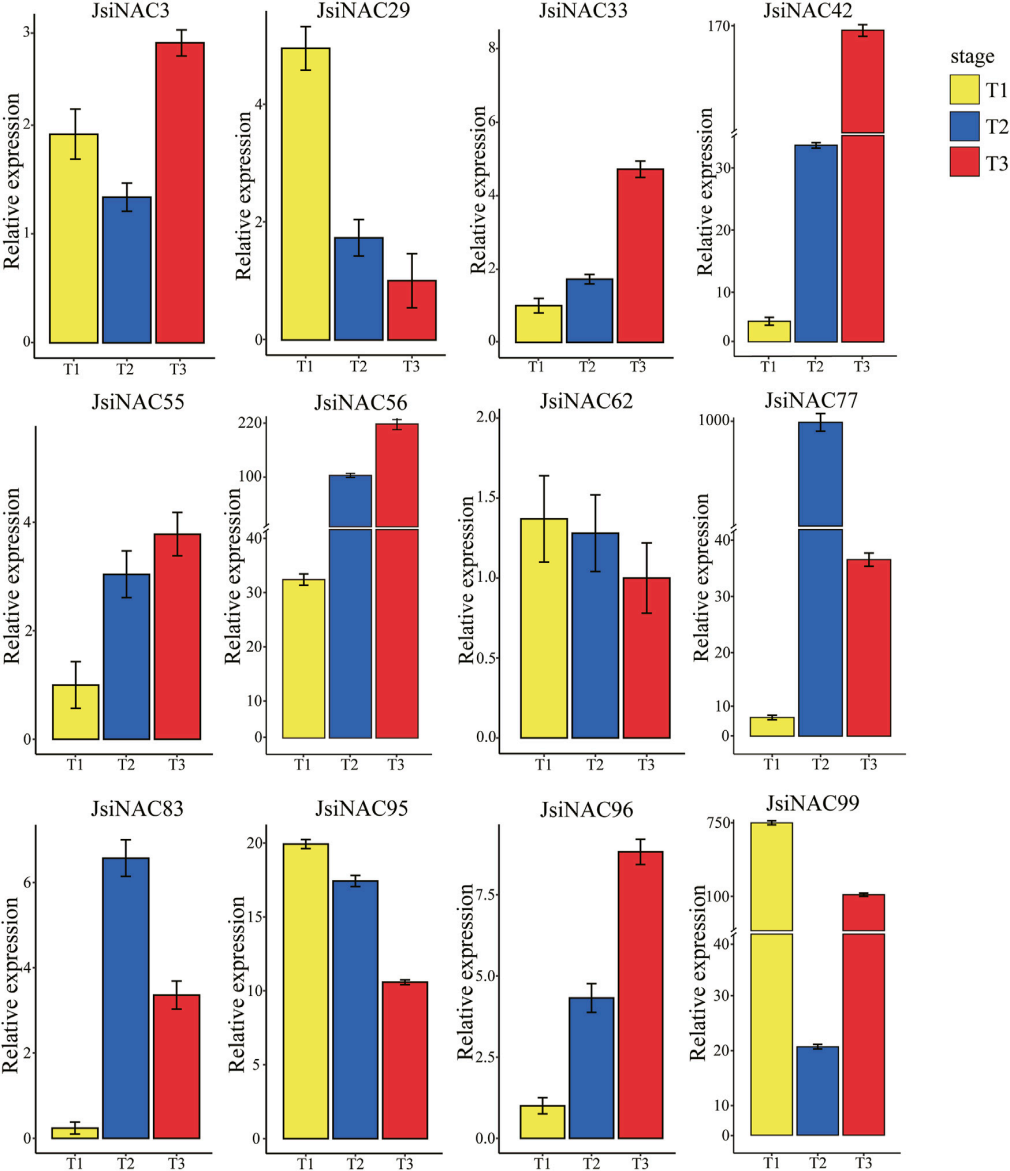
qRT-PCR analysis of 12 *JsiNAC* genes specifically and differentially expressed in the endocarp of iron walnuts at different developmental stages. The *x*-axis represents three developmental periods, T1, T2, and T3 representing 60, 90, and 120 DAP of endocarp in iron walnut “Tie,” respectively. The *y*-axis represents the relative expression levels of each gene, and the values are obtained from qRT-PCR experiments. The data are shown as means of three replicates.

To further explore whether *JsiNAC* genes play a role in endocarp formation in different varieties iron walnuts and to verify the accuracy of the FPKM values obtained from the transcriptome analysis, we performed qRT-PCR analysis of 12 specific *JsiNAC* genes. The expression patterns of these genes were measured at three different developmental stages (T1, T2, and T3) in the endocarp of “Tie” using qRT-PCR, as shown in Figure 5. We found that *JsiNAC29/62/95/99* were more highly expressed in early stage (represented by T1), than decreased in middle and late stages, represented by T2 and T3, respectively. In particular, *JsiNAC95* and *JsiNAC99* showed highest expression in the early endocarp developmental stage, and we speculated that these genes may be involved in the initiation of the endocarp formation in iron walnut. Notably, *JsiNAC99* was assigned to the M subfamily in phylogenetic tree (Figure 2), which was a key regulator of SCW formation in wood fiber. Its expression was elevated again in the late stage of endocarp development compared to the middle stage, the expression pattern suggests that *JsiNAC99* might play a key role in the endocarp lignification of late stage. However, the other genes selected for qRT-PCR analysis exhibited higher expression levels in the mid- or late-stages of endocarp development than in the early stages. Among them, the mid-stage expression of *JsiNAC3* was lower than the early-stage, and the expression was highest in the late-stage of development. The expression of *JsiNAC77* and *JsiNAC83* was low in early stage, but their expression was highest in the middle stage of endocarp development. The expression of the remaining genes (*JsiNAC33/42/55/56/96*) all showed a gradual increase from early to late stage. Interestingly, *JsiNAC55/56/77/83/95/96* were all endocarp-specific genes as shown in Figure 4A, their expression patterns were similar, and they were all clustered in subfamily I in the phylogenetic tree (Figure 2), suggesting that these genes may play essential roles in the middle and late stages of endocarp development in iron walnut. In general, the expression patterns of qRT-PCR in “Tie” were correlated with the trend of the FPKM values from transcriptome analysis of “Dapao,” except for *JsiNAC83*, *JsiNAC95*, and *JsiNAC99*. Hence, these results demonstrated that the expression patterns of *JsiNACs* were similar in different iron walnut varieties, and the expression levels obtained from RNA-seq data were reliability.

## Discussion

Iron walnut is an important nut and woody oil tree, mainly distributed in southwestern of China. The fruit of walnut is known as a pseudodrupe, and its endocarp is composed of multiple layers of thin-walled tissues with small and tightly arranged cells in the early stage, and then the endocarp tissue is lignified to form the polylobate sclereid cells in the later stage, and finally develops into the hard shell (Antreich et al., 2019). Previous studies have found that the main components of walnut endocarp are lignin, cellulose and hemicellulose, and lignin is the main factor determining the thickness of hard shell (Li et al., 2012). Several NAC and MYB transcription factors (TFs) have been identified as master regulators of lignin biosynthesis during SCW formation (Nakano et al., 2015). However, the molecular mechanism governing lignin biosynthesis in the endocarp of iron walnut remains unelucidated. High throughput sequencing technologies and the availability of genome data facilitated genome-wide NAC members identification and characterization in diverse plants, for example, 105, 74, 140, 163 and 142 NAC members have been found in Arabidopsis*, Vitis vinifera, Oryza sativa, Populus trichocarpa,* and *Actinidia eriantha,* respectively (Ooka et al., 2003; Fang et al., 2008; Hu et al., 2010; Wang et al., 2013; Jia et al., 2021). The large number variation of *NAC* genes among plant species suggested that they may have expanded extensively during the evolution of higher plants. NAC TFs have also been studied in different walnut species, such as 121 and 114 *NAC* genes had been identified in *J. regia* and *Juglans mandshurica* (Kang et al., 2021; Li et al., 2021)*.* The number of NAC TFs identified in this study (117 *JsiNACs*) was nearly similar to the NAC gene number in other walnut species, indicating that the number features of NAC genes is stable within the genus Juglans. Numerous NAC TFs have various important functions in plant growth and development, and recent researches reveal that NAC TFs are involved in coping with various biotic or abiotic stresses (Hegedus et al., 2003; Jensen et al., 2008; Wang, et al., 2009; Tran et al., 2010). It is worth noting that lignification of SCWs in plants is not only important for providing rigidity and strength to support cells and plant bodies, but also can increase their resistance to disease through lignification, such as the *TaNAC032* not only regulates lignin biosynthesis genes, also could resist combat Fusarium head blight in wheat (Soni et al., 2021). Lignin may be an important intermediate in regulating antiviral defense via *LrNAC35*, the expression level of *LrNAC35* in *Lilium regale* can be significantly increased after infection with multiple viruses, and the lignin accumulation was enhanced in the cell wall (Sun et al., 2019). Currently, little information is available regarding the functional roles of *NAC* genes in the shell-hardening process of nut fruits, such as walnut, macadamia nut, almonds, and some other nut species.

To comprehensively predict the function of 117 *JsiNAC* genes in *J. sigillata*, we integrated these functionally characterized NAC TFs from Arabidopsis and common walnut to construct the phylogenetic tree (Figure 2). Combining with the conserved motif analysis (Figure 1), we can infer that those phylogenetically closely related NAC TFs within the same subfamily trend to have similar motif compositions, and these orthologous genes are functionally similar. For example, the number of *NAC* genes in I subfamily was the largest in the phylogenetic tree, and the functions of these genes were linked with the development of xylem vessels (*XND1*, *AT5G64530.1*), ovule integument (*NARS1/2*, *AT3G15510.1/AT1G52880.1*), water transport capacity (*NAC025*, *AT1G61110.1*), leaf senescence (*ANAC072*, *AT4G27410.2*) and stress responses (*ATAF2*, *AT5G08790.1*) (Xie et al., 2000; Kunieda et al., 2008; Hickman et al., 2013; Niu et al., 2021; Zhong et al., 2021). In the present study, expression analyses revealed that most *JsiNAC* genes in I subfamily were constitutively expressed in bud, root, fruit, endocarp and stem xylem, implying that they may play important roles in different tissues of the iron walnut plant development. Combining gene co-expression with the phylogenetic tree, *JsiNAC42* displayed high expression in endocarp tissue and its expression levels gradually increased with walnut fruit development. We considered *JsiNAC42* as a hub gene of endocarp lignification, and its homologs *AtNAC1 (AT1G56010.1)* in Arabidopsis worked downstream of TIR1 to transduce the auxin signal for lateral root development (Xie et al., 2002). This results suggested that *JsiNAC42* plays a key role in walnut endocarp SCW regulatory network, and its function might be induced by auxin. Moreover, *JsiNAC77* and *JsiNAC96* specifically displayed high expression in xylem region of walnut stem, while *JsiNAC55, JsiNAC56, JsiNAC83*, and *JsiNAC95* not only exhibited tissue-specific expression in endocarp, but also in stem xylem region, which suggested the function of *JsiNAC* family members in regulating SCW thickening is conservative during plant growth and endocarp development in walnut. Previous studies have offered experimental support for regulating secondary wall biosynthesis for *AtNST1-3* in M subfamily, *AtVNDs* in N subfamily, and *AtSND2-3* in C subfamily of the phylogenetic tree. Especially, the homologs of *NST1* played key roles in the lignified seed coat formation of pumpkin and pomegranate (*Punica granatum*) (Xia, et al., 2019; Lyu et al., 2022). However, most *JsiNAC* except for *JsiNAC52* in the above mentioned three subfamilies showed low levels of expression in both five different tissues and the three endocarp development stages, indicating the distinctive regulator mechanisms between walnut endocarp formation and plant vascular development. Here, we suppose that there might be a new discovery of the NAC TFs function in walnut endocarp formation, which might shed light on walnut as a model for studying shell thickness formation across nut species.

## Conclusion

A total of 117 *JsiNAC* genes were systematically identified and characterized *in silico*, which involves only computational analysis to provide insight into gene function and regulation. These *NAC* genes could be divided into 14 subfamilies, and tissue-specific expression analysis indicated that the *NAC* members in I and J subfamilies were related to endocarp formation and lignification, particularly, *JsiNAC42*, *JsiNAC55*, and *JsiNAC56.* These *JsiNACs* with changed expression during endocarp development were significantly different from SCW related NAC TFs in other species. This research provides a new clue as to how the NAC TFs functions in walnut endocarp formation, and this will facilitate the study of shell thickness formation across nut species.

## Data Availability

The original contributions presented in the study are included in the article/Supplementary Material, further inquiries can be directed to the corresponding author.
