# Supplementary material for: Genomic characterization of the NAC transcription factors, directed at understanding their functions involved in endocarp lignification of iron walnut (Juglans sigillata Dode)
Source: Front Genet. 2023 May 9;14:1168142. doi: 10.3389/fgene.2023.1168142 (PMC10203416; doi:10.3389/fgene.2023.1168142)
Supplement: Supplementary file 2 [file DataSheet4.docx]

Supplementary Material

**Genomic Characterization of the NAC Transcription Factors, Directed at Understanding Their Functions Involved in Endocarp Lignification of Iron Walnut (*Juglans sigillata* Dode)**

**Anmin Yu^1†^, Hanyu Zou^1†^, Ping Li^1^,** **Xiaowei** **Yao^1^, Zekun Zhou^1^, Xu Gu^1^, Rui Sun****^1^****, Aizhong Liu^1*^**

*** Correspondence:** Aizhong Liu: liuaizhong@mail.kib.ac.cn


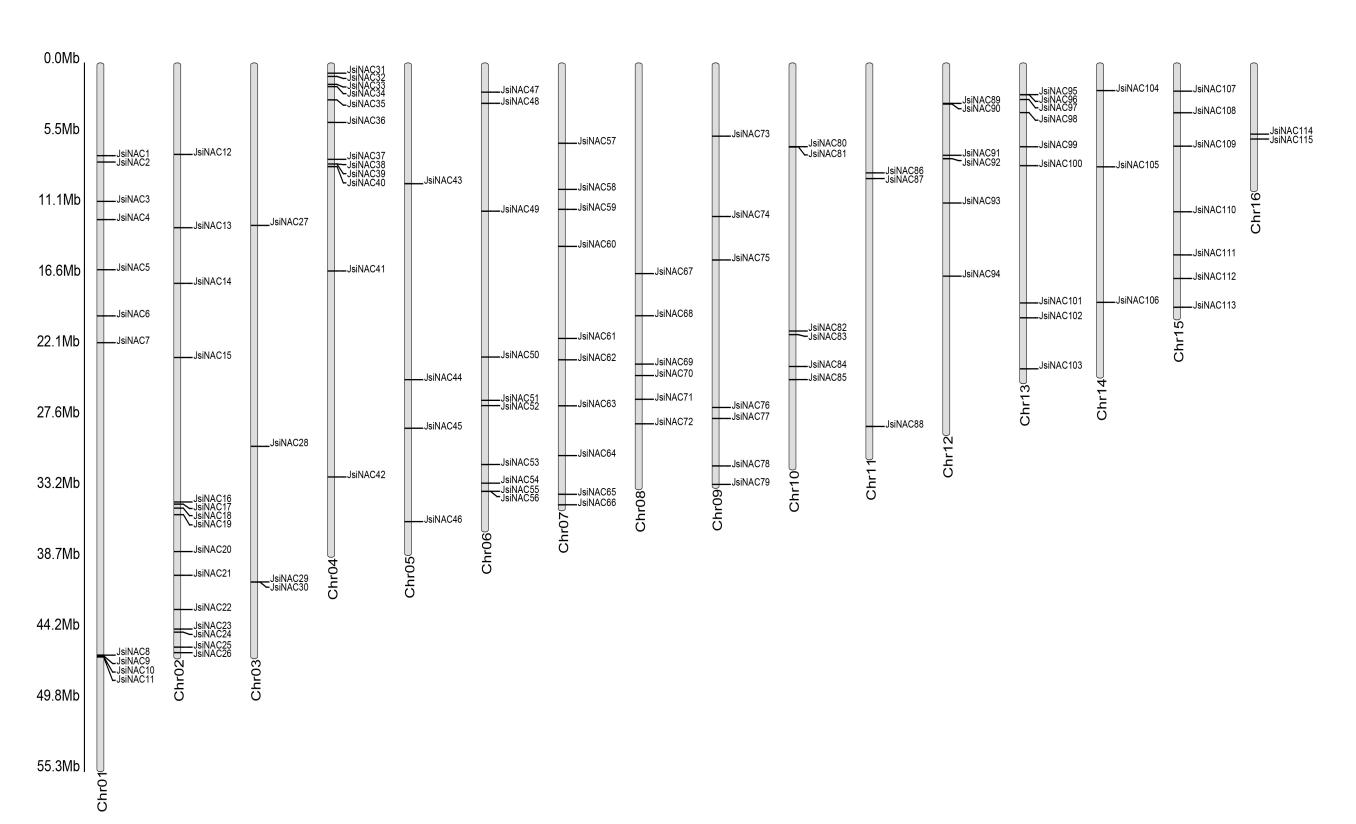
**Supplementary Figure 4. The Distribution of *JsiNAC* Gene on Chromosomes.** The 115 *Jsi*NAC genes are distributed on 16 chromosomes, the gray vertical bars indicate chromosomes, the chromosome number is at the bottom of each chromosome, and the scale on the left represents the chromosome length (Mb).
