# Supplementary material for: Genomic characterization of the NAC transcription factors, directed at understanding their functions involved in endocarp lignification of iron walnut (Juglans sigillata Dode)
Source: Front Genet. 2023 May 9;14:1168142. doi: 10.3389/fgene.2023.1168142 (PMC10203416; doi:10.3389/fgene.2023.1168142)
Supplement: Supplementary file 8 [file DataSheet2.docx]

Supplementary Material

**Genomic Characterization of the NAC Transcription Factors, Directed at Understanding Their Functions Involved in Endocarp Lignification of Iron Walnut** **(*Juglans sigillata* Dode)**

**Anmin Yu^1†^, Hanyu Zou^1†^, Ping Li^1^, Xiaowei Yao^1^, Zekun Zhou^1^, Xu Gu^1^, Rui Sun^1^, Aizhong Liu^1*^**

*** Correspondence:** Aizhong Liu: liuaizhong@mail.kib.ac.cn


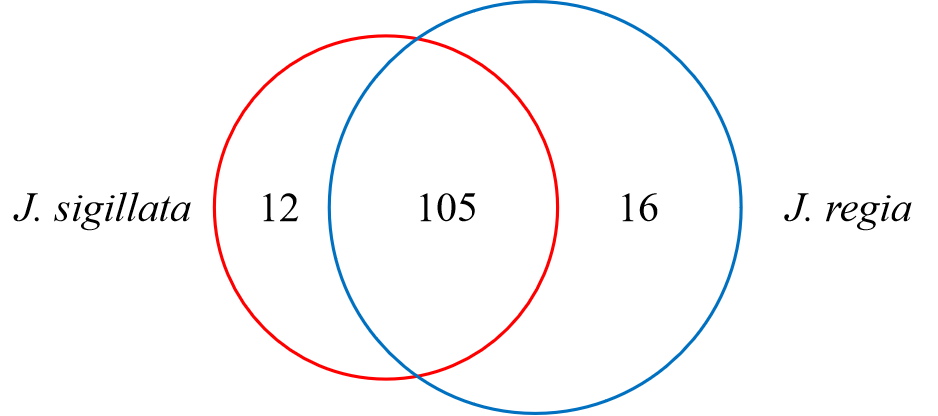


**Supplementary Figure 2.** **The** **105 overlapping genes between *JrNAC* and *JsiNAC* genes.**
