# Supplementary material for: Genomic characterization of the NAC transcription factors, directed at understanding their functions involved in endocarp lignification of iron walnut (Juglans sigillata Dode)
Source: Front Genet. 2023 May 9;14:1168142. doi: 10.3389/fgene.2023.1168142 (PMC10203416; doi:10.3389/fgene.2023.1168142)
Supplement: Supplementary file 10 [file DataSheet1.docx]

Supplementary Material

**Genomic Characterization of the NAC Transcription Factors, Directed at Understanding Their Functions Involved in Endocarp Lignification of Iron Walnut (*Juglans sigillata* Dode)**

**Anmin Yu^1†^, Hanyu Zou^1†^, Ping Li^1^,** **Xiaowei** **Yao^1^,** **Zekun Zhou^1^, Xu Gu^1^, Rui Sun****^1^****, Aizhong Liu^1*^**

*** Correspondence:** Aizhong Liu: liuaizhong@mail.kib.ac.cn


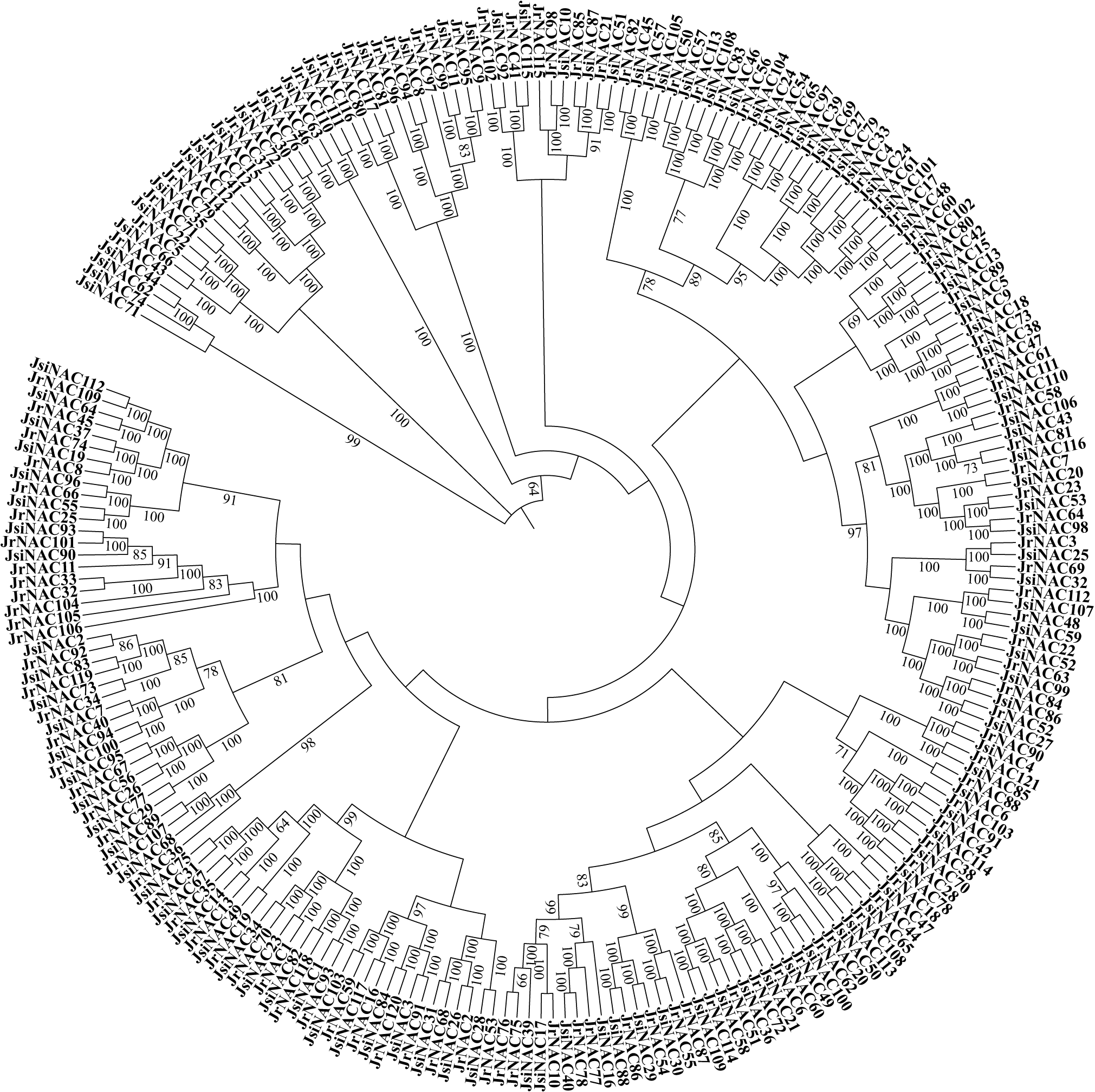


**Supplementary Figure 1. Phylogenetic tree of 121 *JrNAC* and 117 *JsiNAC* genes.** Phylogenetic trees were constructed using the neighbor-joining (NJ) method with 1000 bootstrap replicates.
